# A brain-computer interface for potential non-verbal facial communication based on EEG signals related to specific emotions

**DOI:** 10.3389/fnins.2014.00244

**Published:** 2014-08-26

**Authors:** Koji Kashihara

**Affiliations:** Information Solution, Institute of Technology and Science, The University of TokushimaTokushima, Japan

**Keywords:** neutral faces, source localization, aversive conditioning, face recognition, electroencephalogram, brain computer interfaces

## Abstract

Unlike assistive technology for verbal communication, the brain-machine or brain-computer interface (BMI/BCI) has not been established as a non-verbal communication tool for amyotrophic lateral sclerosis (ALS) patients. Face-to-face communication enables access to rich emotional information, but individuals suffering from neurological disorders, such as ALS and autism, may not express their emotions or communicate their negative feelings. Although emotions may be inferred by looking at facial expressions, emotional prediction for neutral faces necessitates advanced judgment. The process that underlies brain neuronal responses to neutral faces and causes emotional changes remains unknown. To address this problem, therefore, this study attempted to decode conditioned emotional reactions to neutral face stimuli. This direction was motivated by the assumption that if electroencephalogram (EEG) signals can be used to detect patients' emotional responses to specific inexpressive faces, the results could be incorporated into the design and development of BMI/BCI-based non-verbal communication tools. To these ends, this study investigated how a neutral face associated with a negative emotion modulates rapid central responses in face processing and then identified cortical activities. The conditioned neutral face-triggered event-related potentials that originated from the posterior temporal lobe statistically significantly changed during late face processing (600–700 ms) after stimulus, rather than in early face processing activities, such as P1 and N170 responses. Source localization revealed that the conditioned neutral faces increased activity in the right fusiform gyrus (FG). This study also developed an efficient method for detecting implicit negative emotional responses to specific faces by using EEG signals. A classification method based on a support vector machine enables the easy classification of neutral faces that trigger specific individual emotions. In accordance with this classification, a face on a computer morphs into a sad or displeased countenance. The proposed method could be incorporated as a part of non-verbal communication tools to enable emotional expression.

## Introduction

Unlike assistive technology for verbal communication, the brain-machine or brain-computer interface (BMI/BCI) has not been sufficiently established as a non-verbal communication tool for amyotrophic lateral sclerosis (ALS) patients (Nijboer et al., 2009; Tomik and Guiloff, 2010). Although face-to-face communication provides rich emotional information, late-stage ALS patients may experience difficulties in expressing their emotions because the disorder causes severe muscular paralysis (Iversen et al., 2008; Nijboer et al., 2009). Similarly, autistic individuals cannot adequately communicate negative feelings during non-verbal communication (Dalton et al., 2005). Emotions in these individuals will be easily predicted by looking at emotional facial expressions of visitors (e.g., positive feelings triggered by smiling faces and negative feelings stimulated by angry faces). However, people feel certain emotions even when observing an inexpressive face that is associated with an experience or socially relevant memory. Emotional prediction induced by inexpressive faces of visitors will necessitate advanced judgment underlain by various brain activities.

A crucial component of smooth communication is the ability to discern emotional states from facial expressions. Previous neuroimaging studies have implicated the fusiform gyrus (FG) and superior temporal sulcus (STS) as specifically involved in face processing activities (George et al., 1999; Leppänen and Nelson, 2009). This finding is supported by evidence that FG lesions in patients with prosopagnosia impair the ability to perceive facial configurations (Sergent and Signoret, 1992; Barton et al., 2002). The neural system for face perception is divided into a core system (the inferior occipital gyrus, lateral FG, and STS) for visual analysis and an extended system [intraparietal sulcus, amygdala (AMG), insula, etc.] for cognitive functioning in attention, mouth movement, facial expression or identity, and emotion (Haxby et al., 2000).

Healthy people easily respond to facial expressions that communicate happiness, sadness, and anger by using similar facial expressions. Although a patient with severe ALS cannot effectively form facial expressions, family members and carers could, to a certain extent, notice the patient's emotions from facial expressions in visitors. That is, the emotions in the patient will similarly correspond with the facial expressions at which he/she is looking (Keltner and Ekman, 2000). However, a more difficult requirement in coping with social situations is the ability to read an individual's emotional responses to neutral faces. In particular, a person may feel certain emotions when encountering neutral expressions that are associated with previous experiences. However, ALS or autistic individuals cannot directly express their emotions—a situation that diminishes their quality of life. For individuals who are unable to summon appropriate facial expressions during communication, desirable technologies are passive or affective BCIs (Nijboer et al., 2009; Zander et al., 2010) that enable the communication of specific emotions. Such technologies should facilitate real-time reception of and response to patients' emotions rather than be restricted to interpreting facial expressions.

Even a neutral face associated with previous information or memory (e.g., previously observed behavior and personal characteristics, familiar situations, etc.) can elicit various emotions (Todorov et al., 2007) and enhance brain activities (Kleinhans et al., 2007; Taylor et al., 2009). Experimental conditioning studies indicated that specific cues (e.g., angry or fearful faces) associated with negative feelings modulate AMG activity (Morris et al., 2001; Knight et al., 2009). Despite the progress made in research, however, a comprehensive conditioning study on inexpressive faces associated with negative emotions has not been conducted, with specific focus on the rapid neural dynamics (e.g., <1 s) in human cortex networks.

Although the effects of conditioned neutral faces remain unknown, previous studies on electromagnetic brain activity revealed significant electrical responses to faces at 100-ms (P1) and 170-ms (N170) latencies. P1 amplitudes in occipital regions are facilitated by visual stimuli, such as fearful faces (Pourtois et al., 2004). Specific N170 responses are characterized by posterior temporal negative deflection (Bentin et al., 1996; Eimer, 2000) and are slightly but more significantly enhanced in response to fearful faces than to other facial expressions (Batty and Taylor, 2003; Pegna et al., 2008). These face-specific amplitudes may therefore vary even when an individual looks at negatively conditioned inexpressive faces because of the modulation of emotional valances on the basis of individual experience.

Event-related potential (ERP) amplitudes for face stimuli have not been adequately elucidated. For example, some researchers showed that both famous and unfamiliar faces elicit identical amplitudes (Eimer, 2000; Schweinberger et al., 2002); they argue that late-period processing (e.g., 300–500 ms) is more important than early-period processing in the recognition of facial expressions or movements. By contrast, other studies revealed that familiar faces can enhance N170 amplitudes (Rossion et al., 1999; Caharel et al., 2002). In clarifying the reasons for the incongruence in results, a potentially promising approach is applying source localization to total channel responses to determine novel ways of explaining neuronal dynamics in the cortex, even under slightly differing EEG responses between experimental conditions.

Source localization supports the enhanced activation of the lateral FG at P1 and N170 latencies, such as the activation observed in the direct perception of facial expressions (Utama et al., 2009) and face processing during imaginary situations (Ganis and Schendan, 2008). Nevertheless, in the brain's neural response to inexpressive faces giving rise to negative emotions, definitive conclusions regarding the effects of source localization remain elusive. The primary purpose of this study, therefore, was to investigate the effects of aversively conditioned neutral faces on rapid central responses and evaluate the potential of electroencephalogram (EEG) signals as tools for detecting such responses. Source localization was performed to identify cortical activities. The emotions evoked by individual experience with a specific person may cause changes in physiological characteristics during rapid face processing.

If EEG signals can detect actual emotional responses to specific inexpressive faces, the results could be incorporated into the design and development of BMI/BCI-based non-verbal communication tools. Brain signals, such as EEG, magnetoencephalography (MEG), near-infrared spectroscopy, and electrocorticogram (ECoG) data, can be integrated with BMI/BCI functionality, thereby enabling the development of technologies that offer advantages to paralyzed patients (Lebedev and Nicolelis, 2006). In particular, ECoG or intracortical single-unit recordings show high spatial resolution and good signal-to-noise (S/N) ratio. Despite the advantage of this method, however, the invasive nature of the measurement may present technical difficulties and pose clinical risks (Leuthardt et al., 2004). By contrast, EEG or MEG signals can be non-invasively measured regardless of low spatial resolution and the presence of artificial noise (Gramfort et al., 2010). To improve poor S/N ratios, researchers have applied a wavelet transform with reasonable noise reduction to the single-trial classification of EEG signals (Tallon-Baudry et al., 1996; Hsu and Sun, 2009). EEG signals are also convenient for everyday measurement because EEG equipment is inexpensive and portable.

Because substantial changes in the EEG signals measured by scalp electrodes can be easily detected, extensive brain activity in the cortex (e.g., P300 responses) has been successfully employed in active BMI/BCI studies (Piccione et al., 2006; Mak et al., 2012). For non-verbal communication, the FG and STS regions are specifically involved in face processing (George et al., 1999; Hoffman and Haxby, 2000). For example, electrodes placed around posterior temporal regions can detect strong electrical responses to human faces, with the responses exhibiting 100-ms (P1) and 170-ms (N170) latencies (Bentin et al., 1996; Eimer, 2000). Especially for ALS patients who cannot freely move or speak, an important component of smooth communication is the ability to convey the emotions triggered by facial recognition to support persons. In this regard, therefore, real-time BMI/BCI-based face recognition is a desirable next-generation application of EEG signals for the facilitation of non-verbal communication.

Support vector machines (SVMs) are useful and efficient methods for classifying biological signals (Lotte et al., 2007). However, a few problems are presented by parameter settings for SVMs that are directly linked to accuracy. To consider approaches to the use of BMI/BCIs with EEG signals, an essential requirement may be the abstraction of brain activity at functional frequencies under reduced artificial noise (Tallon-Baudry et al., 1996). Such issues are effectively addressed by time–frequency analyses (Kashihara et al., 2009), which could also elucidate the neural activities that are crucial for face processing. The time–frequency data for an SVM classifier enable the efficient extraction of meaningful changes in ERP responses. Accordingly, the second purpose of this study was to develop and evaluate an analytical method in which an SVM classifier evaluates negative emotional responses to inexpressive faces. The method was developed on the basis of time–course and time–frequency EEG data. A face morphing application triggered by the SVM classifier was also tested to determine its utility in non-verbal communication.

## Study 1: EEG measurement

Using EEG signals, this study investigated the effects of aversively conditioned neutral faces on rapid central responses. The emotions induced by individual experience with a specific person may modify the physiological characteristics that arise during face processing.

### Materials and methods

#### Participants

A total of 22 right-handed healthy volunteers from Nagoya University were recruited for the study. The physiological responses of the 12 participants (6 males and 6 females; age: 27.0 ± 0.9 years) to conditioned neutral faces were examined. The remaining 10 participants (see Section Basic Study of Neutral Face Stimuli) were asked to take part in a basic experiment to evaluate the equality of the face stimuli used in this work. All the participants had normal or corrected-to-normal vision and had no history of serious medical problems. This study was approved by the ethics committee of our institute. Written informed consent was obtained from all the participants after they were provided an adequate description of the experiment.

#### Stimuli

Five inexpressive faces, as visual stimuli, were selected from the Japanese Female Facial Expression (JAFFE) database^1^. Thirty scenery images without artificial objects (e.g., skies, mountains, seas, etc.) were collected from free web sources. All the images were converted into grayscale bitmap images. The visual stimuli were presented on a 21-inch CRT monitor (640 × 640 pixels, with a resolution of 1024 × 768 pixels) that was positioned at the same height as the participants' eyes. The distance from the stimuli was set at 140 cm, indicating a visual angle within almost 5°. During the experiments, a loudspeaker was placed behind a participant and a 100-dB white noise burst was used as the auditory stimulus (Morris et al., 1998).

#### Procedure

After the sensors for the EEG measurement were attached onto the participants, they were asked to view a short series of images (10 trials) during the acclimation period. They were first asked to rate three face images, after which the two-phase experiment was initiated. The two phases were (1) aversive conditioning to a neutral face (*conditioning* phase) and (2) physiological measurement using the conditioned stimuli (*data acquisition* phase).

**Figure 1:**
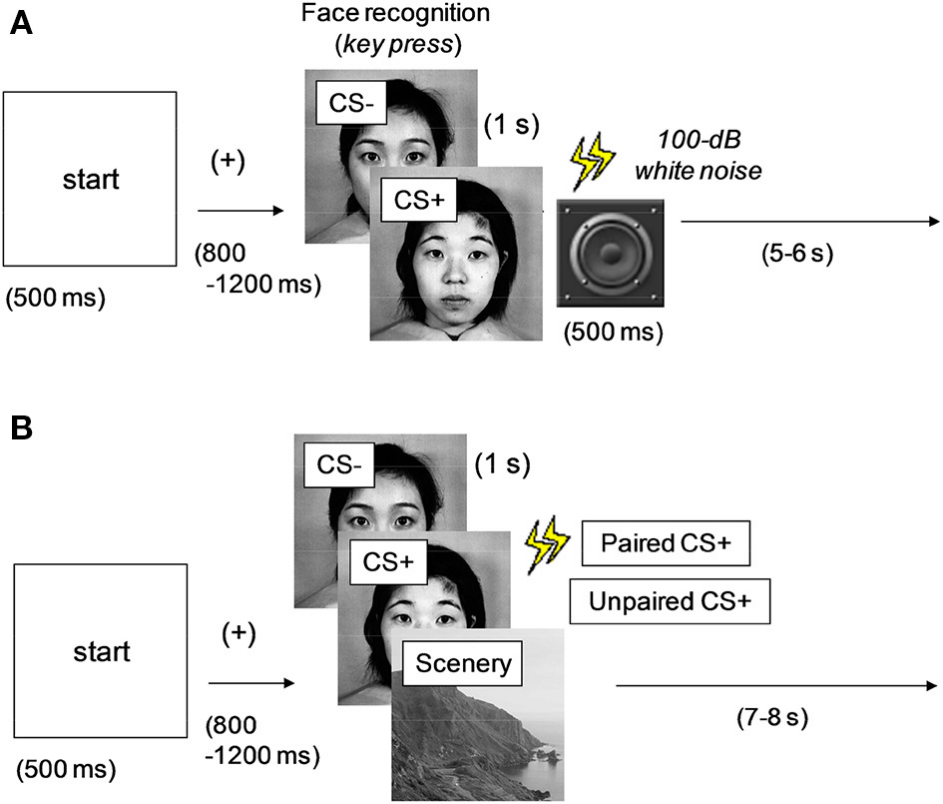
**Experimental procedures in the (A) conditioning and (B) data acquisition phases**. One of the multiple images was presented at every trial. CS+, aversively conditioned face stimuli; CS−, unconditioned stimuli. All the faces were neutral; CS+ was followed by a noise burst in **(A)** (all) and **(B)** (50%).

***Conditioning phase***. Figure 1A shows the experimental procedure adopted in the conditioning phase. A 500-ms “start” stimulus was followed by an interstimulus interval (ISI) between 800 and 1200 ms, after which one of the two faces selected from the dataset was presented onscreen for 1 s. A single neutral face (conditioned stimulus: CS+) was always followed by a 500-ms noise burst; the other face (CS−) was not paired with the noise burst. Fifty percent of all the face stimuli were of CS+ type (or CS−), and the image types were presented in random order. To evaluate the difference between the two faces, the participants were asked to press the left and right keys for the first and second faces, respectively. Intertrial interval (ITI) was varied between 5 and 6 s. This phase comprised 40 trials (20 trials under each stimulus condition). After all the trials were completed and the participants finished their 5-min rest period, the next phase was initiated.

***Data acquisition phase***. Figure 1B shows the experimental procedure used in the data acquisition phase. A 500-ms “start” stimulus was followed by an ISI jittered between 800 and 1200 ms. One of the two faces (CS+ and CS−) used in the conditioning phase or a scenery image (randomly selected from the dataset) was presented for 1 s. The images in the three types (unconditioned and conditioned neutral faces and scenery) were presented in random order. Half of the CS+ images were followed by a 500-ms noise burst (paired CS+), whereas the rest were presented without sound (unpaired CS+). This method was intended to efficiently maintain the effect of aversive or fear conditioning and was based on a procedure discussed in previous studies (Büchel et al., 1998; Morris et al., 2001). The CS− faces were always followed by silence, and the ITI was between 7 and 8 s.

This phase was initiated in three blocks, with each block involving 72 trials (24 trials for each image type). The rest period between the blocks was 5 min. To enable the participants to concentrate on the tasks, they were asked to press a key as quickly as possible when the cue stimulus of a railroad image was presented twice in a block. The participants were asked to refrain from body movements during the physiological measurement and to refrain from blinking during the image presentations. The face images (CS+, CS−, and a dummy) were also rated at the final period of the experiment.

#### Data acquisition

***EEG recording***. EEGs were recorded using EGI Inc.'s HydroCel Geodesic Sensor Net (65 channels) in accordance with the international 10-10 electrode system. The signals from an EEG amplifier (Net Amps 300) were sampled at 500 Hz with data acquisition software (Net Station ver. 4.2). The electrode impedances for all channels were kept below 50 kΩ, as recommended by EGI Inc. The EEG amplifier used in this study can record input with high electrode impedance, without the attachments causing scalp abrasions and without the need for a recording paste and gel. The recording net with electrodes uses a saline solution for the electrical conductor, thereby resulting in high electrode impedances. The high impedances are regulated by the amplifiers to guarantee recording accuracy (Ferree et al., 2001). The features of the EEG amplifier are also highly useful in EEG recordings for patients who cannot withstand lengthy setup procedures or painful scalp abrasions.

***Rating of face stimuli***. The participants were asked to rate neutral faces (0 = not at all, 1 = mild, 2 = moderate, 3 = strong, and 4 = extreme) to evaluate the changes in the emotions that arose [fear, anxiety, aversion, discomfort, anger, relief, favor, and pleasure in relation to social situations (Nesse, 2005)] before and after the experiment. Three images of neutral faces were randomly chosen from the database and presented onscreen; these images were the same as the CS+ and CS− faces and a dummy image that had not been presented in the previous experiments. The participants reported the emotions that they instantaneously experienced when they looked at the presented faces. In the final rating, the degree to which they experienced unpleasant feelings upon exposure to the noise burst (a five-point scale of 0–4) was confirmed by oral declaration.

#### Data analysis and statistics

All data are expressed as mean ± standard error. *p*-values less than 0.05 were considered statistically significant.

***Event-related potentials***. The average reference montage and a digital bandpass filter of 1–30 Hz were applied offline. For all the conditions, the EEG signals were segmented into epochs ranging from 100 ms before stimulus onset to 700 ms after stimulus onset. Baseline correction was performed by subtracting the mean of the 100-ms pre-stimulus interval from the data after stimulus onset. Trials in which ocular activity was greater than ±50 μV within a 50-ms period or movement artifacts with amplitudes exceeding ±200 μV were excluded from analysis. Although noise due to micromovements may contaminate EEG signals, sufficient signal averaging can eliminate as much of this noise as possible. After the trials were averaged in each condition, the grand average among the participants was calculated. Data on cue stimuli with key presses were excluded from the analysis. The regions of interest were the left (P7, P9, TP9) and right (P8, P10, TP10) posterior temporal regions that are correlated with face processing; the bilateral occipital electrodes (O1 and O2) that are related to attention in the primary visual cortex were used in the P1 analysis.

In the ERP responses at the posterior temporal regions, the maximum values between 60 and 120 ms and the minimum values between 100 and 200 ms were defined as P1 and N170 responses, respectively. For every amplitude in the early (P1 and N170) and late (average between 200 and 700 ms under a 100-ms analysis window) ERP components, repeated Two-Way ANOVA [two levels (left and right) in the bilateral recording sites; three levels (CS+, CS−, and scenery) in the presented images] was performed. In significant main effects, the Holm method was used for multiple comparisons.

***Source localization***. Standardized low-resolution electromagnetic tomography (sLORETA, the LORETA-KEY software package) was used for source localization, which has a possibility to estimate the local region from which cortical generators originate in each time window by solving the inverse problem (Pascual-Marqui, 2002). Because sLORETA analysis depends on noise levels (Grave de Peralta Menendez et al., 2009), the EEG data obtained after sufficient signal averaging were applied in the source localization. The solution space of sLORETA is restricted to 6239 voxels with a 5-mm^3^ cortical gray matter.

The average of the post-stimulus period between 50 and 700 ms was compared with that of the baseline (100-ms pre-stimulus period) in each condition. The sLORETA images for the average of the ERP data in the P1 (60–120 ms), N170 (120–150 ms), and post-stimulus periods between 200 and 700 ms (100-ms time window without overlap: five windows) were then calculated to compare the difference between the CS+ and CS− conditions. The P1 and N170 periods that were analyzed were determined from the results on the ERP responses.

The statistical non-parametric map with smoothing and linear scaling (Nichols and Holmes, 2002) was used to estimate the significantly activated parts determined from the source localization between the CS+ and CS− conditions. Voxel-by-voxel *t*-tests of the LORETA images were performed. The significance threshold was based on a permutation test (6000 rounds). The corrected *t*-values were plotted onto a magnetic resonance imaging (MRI) template (Colin27 brain, T2-weighted images) with a color scale bar. As an experimental limitation, the correct location may have been slightly shifted because a standard brain template (Colin27 brain) was used for each subject. All the *p*-values were one-tailed. Results are presented in the Montreal Neurological Institute coordinates with assigned Brodmann's area labels.

***Rating of face stimuli***. The first rating scores were subtracted from the final scores. A positive value indicates increased appeal and a negative value indicates the opposite. The extent to which the participants experienced unpleasant feelings upon exposure to the noise burst was calculated as the average across all the participants.

For the changes in rating scores before and after the conditioning experiment, repeated Two-Way ANOVA (three levels of CS+, CS−, and dummy images and eight levels of basic and social emotions) (Nesse, 2005) was carried out under the assumption of an equal-interval scale. For significant main effects, the Holm method was used for multiple comparisons.

### Results

#### ERP responses

**Figure 2:**
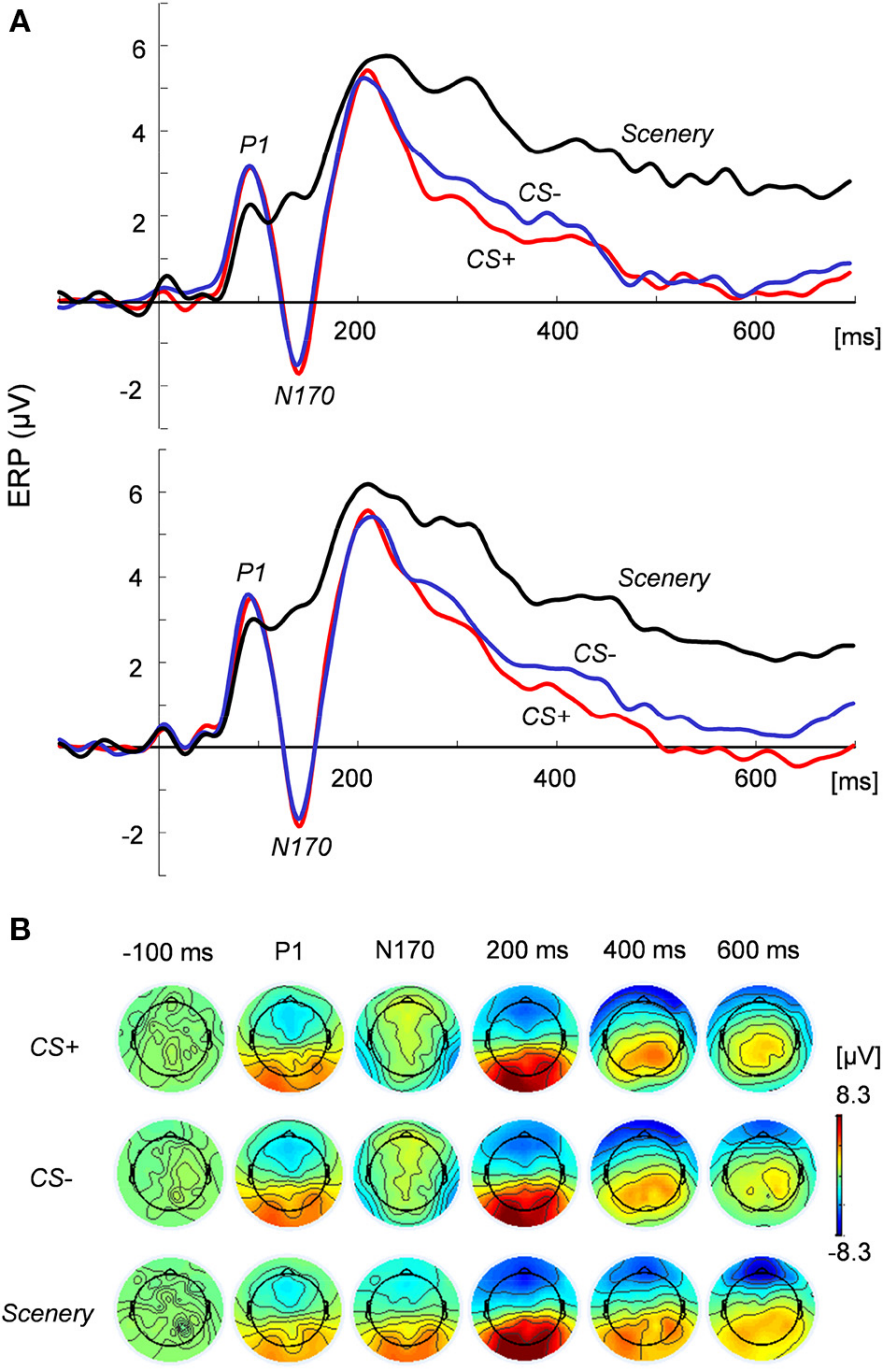
**ERP responses to and topography of the presented images. (A)** ERP responses at the left (*top*) and right (*bottom*) posterior temporal regions under the CS+, CS−, and scenery conditions. **(B)** Two-dimensional topography of the presented images at P1, N170, and latencies of −100 (pre-stimulus), 200, 400, and 600 ms.

Figure 2 shows the grand average of the ERP responses in the (Figure 2A) bilateral posterior temporal regions and (Figure 2B) two-dimensional topography at the focused latencies. At around 90 ms from face stimulus onset, the peak positive potential (P1) appeared, followed by a considerable negative potential (N170) at around 140 ms. Especially for late latency (600 ms in Figure 2B), the topographical maps of EEG activity changed across the image types.

ANOVA revealed the main effects of the image condition in the P1 [*F*_(2, 44)_ = 4.15, *p* < 0.05] and N170 amplitudes [*F*_(2, 44)_ = 63.67, *p* < 0.01] at the posterior temporal lobe. The N170 values for the face images were significantly (*p* < 0.01) greater than that for the scenery image in each hemisphere. However, these values did not significantly differ between CS+ and CS−. For the P1 amplitude at the occipital electrodes (O1 and O2), the main effects and interaction were statistically non-significant.

For the ERP responses between 200 and 700 ms (100-ms time window), ANOVA indicated that type of image exerted significant main effects: *p* < 0.01 in each period. For all the time windows, the average ERP values for the face images in each hemisphere were significantly lower than that for the scenery (*p* < 0.01). Especially in the period between 600 and 700 ms [*F*_(2, 44)_ = 28.72, significant main effect], the CS+ value in the right hemisphere decreased to a more significant extent than did the CS− value (*p* < 0.05).

#### Source localization

**Figure 3:**
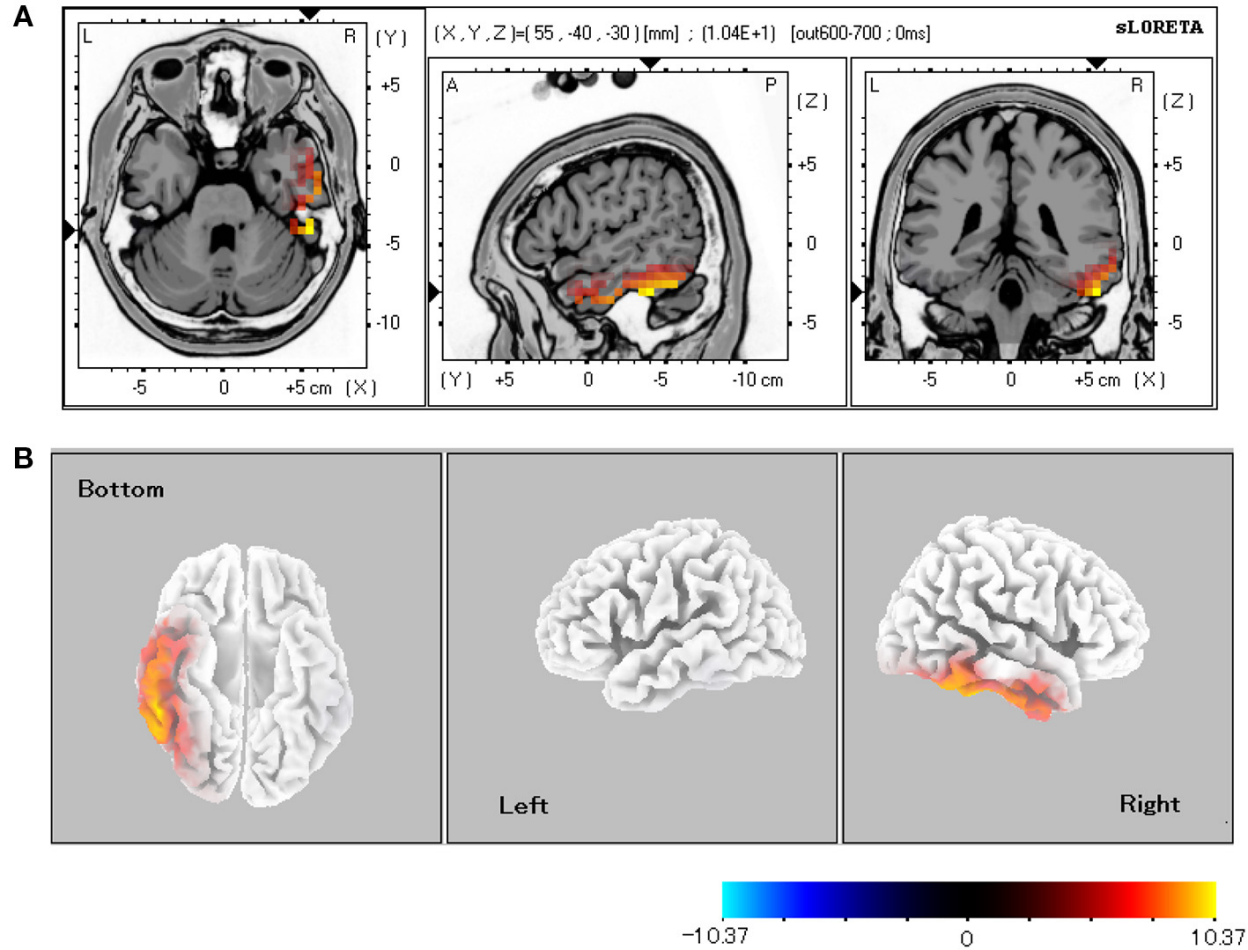
**sLORETA comparison of source localizations between the CS+ and CS− conditions. (A)** MRI planes (Colin27 brain) in the axial, sagittal, and coronal views and **(B)** cortical maps from the bottom, left, and right sides of the average among the 600–700-ms latencies (CS+ vs. CS−). The statistically significant regions acquired from all the subjects were displayed on a standard brain template in **(A)**. The source estimation indicates the significant roles (*p* < 0.05 for all the subjects) of the right lateral fusiform gyrus.

Table 1 shows the statistically significant regions among all the participants (baseline vs. post-stimulus response) in each experimental condition. In relation to the baseline, the significant areas localized by sLORETA (*p* < 0.05) were the bilateral FG, inferior temporal gyrus, and middle temporal gyrus for both the CS+ and CS− conditions. Especially in the CS+ condition, the right hemisphere exhibited stronger activity than did the left hemisphere. During the 600–700-ms latency, the right FG was more significantly activated under CS+ than under CS− (Figure 3; *p* < 0.05).

**Table 1:** **Brain areas of significant activity (*p* < 0.05) under the face image types in relation to the baseline**.

**Region**	**Side**	**MNI coordinates (mm)**			***t*-value max**	**Voxels**	**BA**
		***x***	***y***	***z***			
**CS+ vs. BASELINE**							
FG	Left	−55	−40	−30	9.5	5	20, 37
	Right	55	−40	−30	13.3	34	19, 20, 36, 37
ITG	Left	−55	−5	−40	10.2	5	20, 37
	Right	60	−35	−25	12.3	36	20, 37
MTG	Left	−	−	−	−	0	−
	Right	60	−45	−20	11.8	21	20, 21, 37, 38
**CS− vs. BASELINE**							
FG	Left	−55	−40	−30	16.7	44	19, 20, 36, 37
	Right	55	−40	−30	15.2	39	19, 20, 36, 37
ITG	Left	−55	−5	−40	17.2	50	20, 37
	Right	60	−35	−25	14.1	38	20, 37
MTG	Left	−60	−45	−20	15.5	31	20, 21, 37, 38
	Right	60	−45	−20	13.6	20	20, 21, 37, 38

*CS+, conditioned stimulus; CS−, unconditioned stimulus. BA, Brodmann's areas; FG, fusiform gyrus; ITG, inferior temporal gyrus; MTG, middle temporal gyrus*.

#### Rating of face stimuli

**Figure 4:**
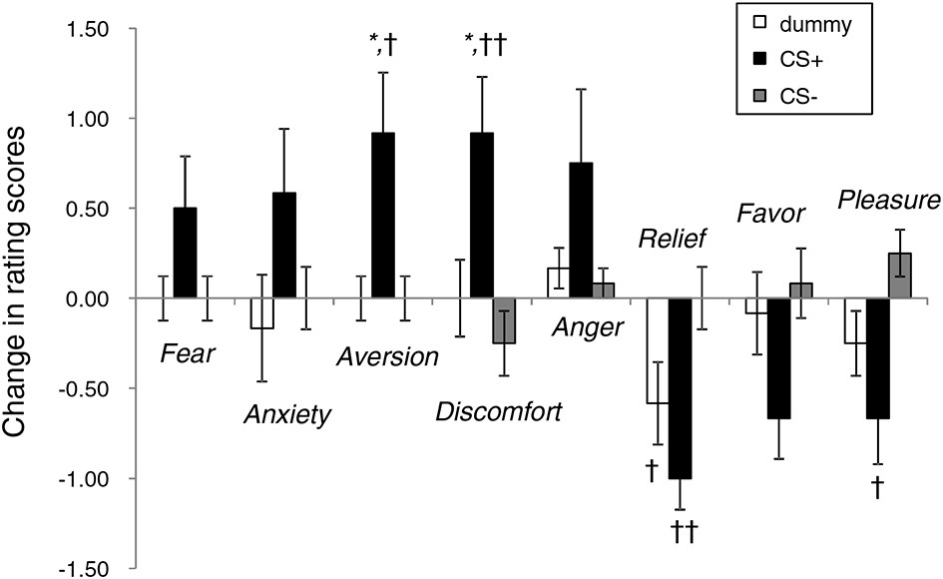
**Changes in the rating scores of emotional responses to neutral face stimuli before and after the conditioning experiments**. ^*^p < 0.05 vs. a dummy; ^††^*p* < 0.01, ^†^*p* < 0.05 vs. CS−.

Figure 4 illustrates the affective changes found under the three face conditions. Overall, the rating scores for CS+ tended to reflect increased negative emotions and decreased positive emotions. ANOVA revealed the main effect of emotion [*F*_(7, 252)_ = 6.17, *p* < 0.01] and significant interaction [type of image × emotion, *F*_(14, 252)_ = 4.54, *p* < 0.01]. In the items showing a significant simple main effect, the CS+ and CS− conditions significantly differed in the rating scores for aversion (*p* < 0.05), discomfort (*p* < 0.01), relief (*p* < 0.01), and pleasure (*p* < 0.05). The changes in the scores for aversion (*p* < 0.05) and discomfort (*p* < 0.05) in the face under CS+ were significantly higher than those derived when the dummy was presented. The extent to which unpleasant feelings were experienced upon exposure to the noise burst was 3.0 ± 0.3 (75% of the max. score).

### Basic study of neutral face stimuli

The same procedure as that applied in the data acquisition phase (see Section Procedure) was performed to evaluate the effect of neutral face stimuli under no condition (7 males and 3 females; age: 25.0 ± 0.7). The ITI was between 5 and 6 s because no aversive conditioning was applied. The parameters for the ERP responses (P1, N170, and latencies between 200 and 700 ms) and the significant source localization sites were statistically evaluated. The neutral faces exhibited no significant differences in terms of ERP response amplitudes and source estimation (*p* > 0.05), indicating equality across the image dataset.

### Discussion 1

Even for the inexpressive faces, aversive conditioning in which the noise burst was used induced a posteriori negative feelings. Although greater attention was paid to the faces than to the scenery, the CS+ and CS− conditions exhibited statistically non-significant differences in the N170 amplitudes in the posterior temporal regions and the P1 amplitudes at the O1 and O2 sites. This result indicates that an extensive and meaningful process of distinguishing the differences between the inexpressive faces would have worked, except in the early face processing. By contrast, the late ERP response (600–700 ms) evoked by the aversively conditioned neutral faces resulted in a statistically significant ERP component at the electrodes attached around the posterior temporal lobe; this result suggests the involvement of higher face recognition functions (Eimer, 2000) under situations wherein expressions are associated with a previous negative experience. A limitation of the experiment was that the meaningful electrode positions were carefully established to enable the detection of EEG responses. Nevertheless, other brain functions may be detected or missed, depending on region of interest. Severe ALS patients may also cause attenuated brain activity (Guo et al., 2010).

For facial expression or mental imagery, previous studies involving source estimation indicated that some brain areas, including the FG, are activated for early face processing at P1 and N170 (Ganis and Schendan, 2008; Utama et al., 2009). Even for the inexpressive faces with aversive conditioning, the source estimation in this study indicated continuous FG activation in both the CS+ and CS− conditions (Table 1). Furthermore, the right FG was significantly activated, with longer latencies of 600–700 ms under CS+ than under CS− (Figure 3). This result presumably reflects aversively conditioned responses to incoming noise burst.

The AMG is activated by short-term fear or aversive conditioning (Büchel et al., 1998; Morris et al., 2001; Knight et al., 2009), and viewing emotional faces effectively increases the connectivity between the AMG and FG (Leppänen and Nelson, 2009). The mental imaging of a face can also enhance FG activity (Ganis and Schendan, 2008). These findings suggest that recalling a conditioned face that reflects negative emotions similarly activates the FG and AMG. Because the results of the present study (Table 1, Figure 3) showed strong activation of the inferior temporal cortex (FG) rather than the STS, negative learning may have been performed via the inferior network for face recognition, through which the AMG and FG were accessed (Morris et al., 1998; Knight et al., 2009). The STS is generally activated by the recognition of gaze direction or facial expressions of emotion (Haxby et al., 2000). Because the visual targets (i.e., inexpressive faces) of the aversive conditioning in this study did not feature such dynamic movements, the STS activity may not have influenced conditioned facial perception and/or recognition.

The central responses were modulated primarily at the right sides of the FG; this modulation is related to the aversively conditioned neutral face stimuli. The activated area (Table 1) on the left-side FG (vs. the right side) under CS+ decreased to 1/7 (5 vs. 34 voxels), whereas the ratio under CS− (44 vs. 39 voxels) generally remained at around 1. This result presumably reflects the intensive recruitment of right-side neural functions in predicting impending danger. This finding is supported by previous studies in which the right-side FG activity in patients with prosopagnosia was dominant (Barton et al., 2002); this activity also dominated even during presentations of a fearful face to healthy participants (Pegna et al., 2008).

The aversion and discomfort reflected by the emotional ratings were significantly increased by the conditioned neutral face (Figure 4), which could trigger changes in brain activity. By contrast, the fearful emotion was non-significantly correlated with the conditioned neutral faces, which would have failed to induce the ERP responses (i.e., early face processing responses, such as P1 and N170) specific to typical fear stimuli (Batty and Taylor, 2003; Pegna et al., 2008). Note, however, that the participants in this study may have experienced unconscious fear during the EEG measurement.

For ERP studies that focus on a specific stimulus, sLORETA software is equipped with a procedure for baseline correction, which has been applied in numerous studies on source localization (e.g., Utama et al., 2009; Scharmüller et al., 2011). Nevertheless, baseline correction must be carefully used in source localization given the occurrence of local changes in each sensor location (http://www.electrical-neuroimaging.ch/faq.html).

The mathematical constraint in LORETA software is the smoothness of spatial activation, in which neighbor neurons are assumed to exhibit similar activations. From direct measurement on neural tissue, active states are characterized by a reduction in the synchrony of adjacent neurons (Cruikshank and Connors, 2008; Poulet and Petersen, 2008). Contrary to the LORETA hypothesis, sluggish states may be identified by the similarity in activity of neighbor neurons under a defined time window for source localization (Grave de Peralta Menendez and Andino, 2000).

The conditioned faces further emphasized activity in the right FG (i.e., face-selective cells) during late facial processing. The EEG equipment detected responses at the posterior temporal regions; these responses reflect activity in face-responsive neurons. Neutral stimuli other than faces would affect various brain areas with different EEG responses. Expert object recognition may stimulate the same face-selective cells (Tanaka and Curran, 2001), although such recognition is a rare response.

## Study 2: application of a brain-computer interface

Study 1 revealed that the posterior temporal lobe ERP responses to the aversively conditioned neutral faces significantly changed during late face processing. As previously stated, this study developed an efficient method for classifying implicit negative emotional responses to specific neutral faces by using EEG signals.

### Materials and methods

#### Classification by SVM

The SVM classifier for determining a hyperplane that optimally separates samples from two classes with the largest margin (Cortes and Vapnik, 1995; Cristianini and Shawe-Taylor, 2000) was used in this study. An optimal SVM separating hyperplane is calculated by solving constrained optimization thus:

(1) $$\underset{z,b,\xi }{\min}\left(\frac{1}{2}{\left\|z\right\|}^{2}+C\sum_{i=1}^{l}\xi_{i}\right).$$

Equation (1) is subject to *y_i_* (***z***· ϕ (***x***_*i*_) + *b*) + ξ_*i*_ ≥ 1 and ξ_*i* ≥ 0_ (*i* = 1, ···, *l*), where *l* is the number of training vectors, *y_i_* ∈ {−1, +1} denotes the class label of the output, and ‖***z***‖^2^ = ***z***^*T*^ ***z*** represents the squared Euclidean norm. Weight parameter *z* determines the orientation of the separating hyperplane, *b* is a bias, ξ_*i*_ is the *i*th positive slack parameter, and ϕ shows a non-linear mapping function. Parameter *C* indicates the penalty term. With a large *C*, a high penalty is assigned to training errors. The two points closest to the hyperplane substantially affect the orientation, thereby resulting in a hyperplane that is close to other data points. With a small *C*, these points move inside the margin and the orientation of the hyperplane changes, thereby generating a large margin. To address this issue, a formulation of the SVM that uses the parameter 0 < ν ≤ 1 can be applied. This parameter can regulate the fractions of support vectors and margin errors (ν-SVM).

The vector ϕ(***x***_*i*_) that corresponds to a non-zero value is a support vector of the optimal hyperplane. A desirable approach is to use a small number of support vectors to complete a compact classifier. The optimal separating hyperplane is calculated as a decision surface of the form sgn:

(2) $$f(x)=\text{sgn}\left(\sum_{i=1}^{L}\alpha_{i}y_{i}K(x_{i},x)+b\right),$$

where sgn(.) ∈ {−1, +1}. *K* is the non-linear kernel function, and it projects samples to a high-dimension feature space via a non-linear mapping function. *L* is the number of support vectors. As the non-linear kernel, the radial basis function is defined as

(3) $$K(x_{i},x_{j})=\exp\left(-\gamma ||x_{i}-x_{j}|{|}^{2}\right),$$

where the value of the kernel parameter γ determines the variance of the function.

#### Features for SVM

**Figure 5:**
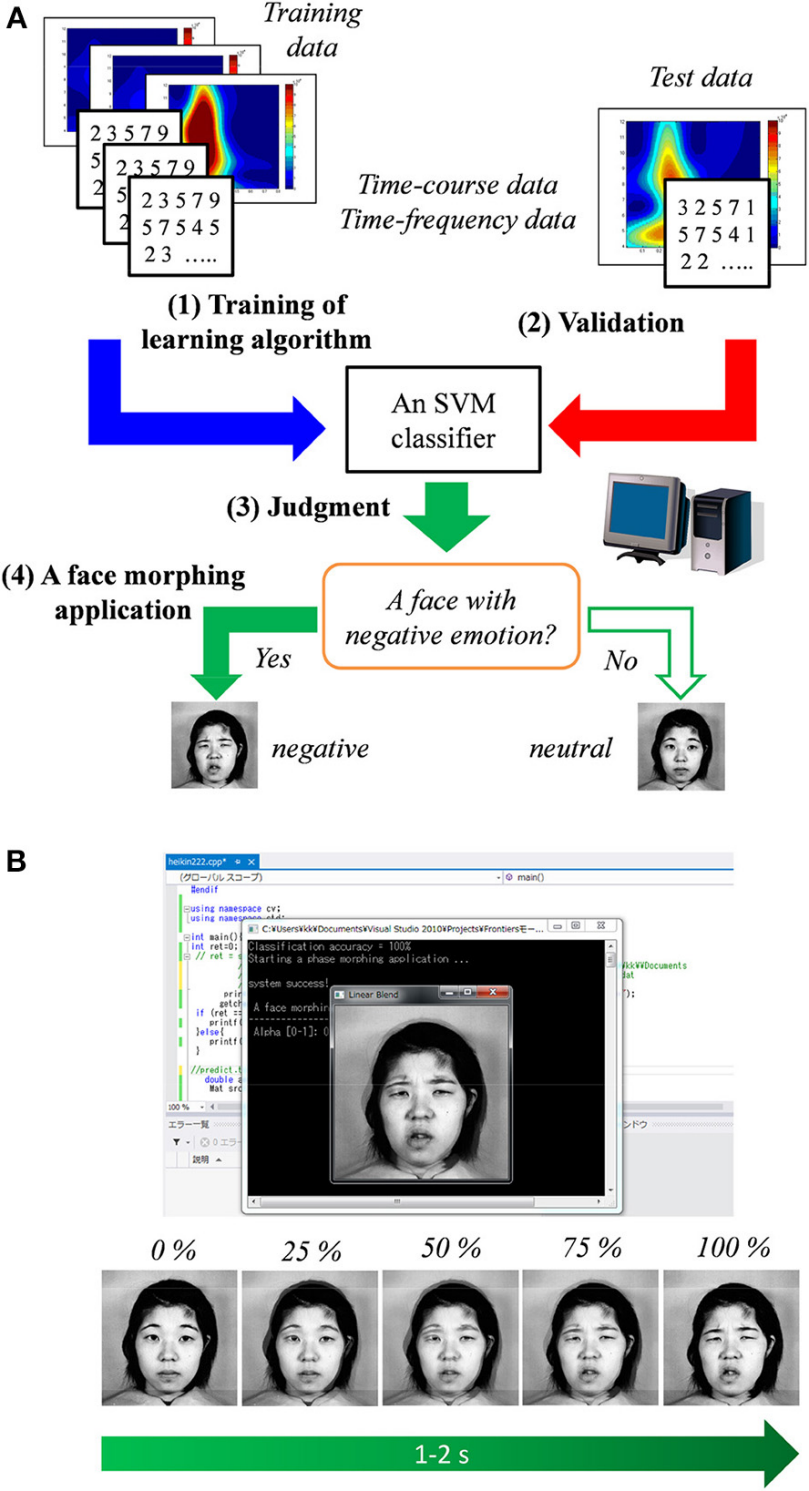
**(A)** Diagram of the proposed method in which a support vector machine (SVM) classifier is used. **(B)** Illustration of the face morphing software triggered by the classifier's result.

Figure 5A shows the diagram of the BCI based on the SVM classifier and face morphing application. Time–course and time–frequency data for the SVM classifier were extracted from the ERP responses to the face and scenery images.

- Time–course data: The time–course data that averaged between 600 and 700 ms at the right (P8, P10, TP10) posterior temporal regions were abstracted as the SVM features. These data correspond to the considerable change in the ERP responses to visual stimuli (Figure 2A).
- Time–frequency data: The time–frequency data obtained from the wavelet transform of raw data were regarded as the SVM features because raw signals can contaminate external noise. The EEG signals were convoluted by the complex Morlet wavelet (Tallon-Baudry et al., 1996; Kashihara et al., 2009) as follows:
  (4) $$w(t,f_{0})=\exp\left(\frac{-t^{2}}{2\sigma_{t}^{2}}\right)\cdot \exp(2\pi f_{0}it)/\sqrt{\sigma_{t}\sqrt{\pi }}.$$

**Figure 6:**
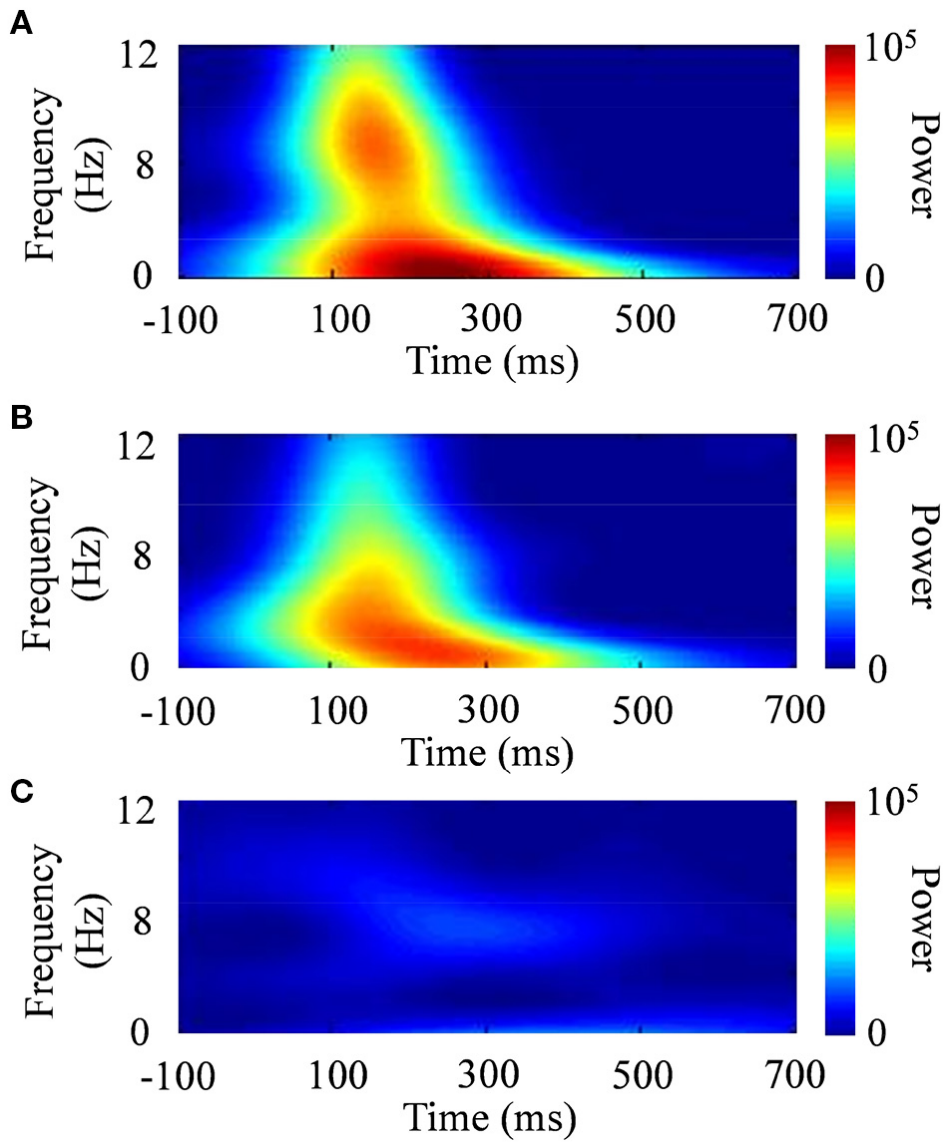
**Typical examples of time–frequency responses after the wavelet transform. (A)** CS+, **(B)** CS−, and **(C)** scenery.

The standard deviation of the time domain (σ_*t*_) is inversely proportional to the standard deviation of the frequency domain [σ_*f*_ = (2π σ_*t*_)^−1^]. The effective number of oscillation cycles in the wavelet (*f*_0_/σ*f*) was set at 6, with *f*_0_ ranging from 4 to 12 Hz (i.e., theta and alpha bands) in increments of 0.1 Hz. After the subtraction of a linear trend, the continuous wavelet transform of a time series [*u*(*t*)] was calculated as the convolution of a complex wavelet with *u*(*t*): *ũ*(*t, f*_0_) = *w*(*t, f*_0_)*u(*t*). The squared norm of the wavelet transform was calculated in a frequency band at around *f*_0_. Figure 6 shows examples of the wavelet transform in the ERP responses to the presented images. The power spectrum of the wavelet transform in the face stimulus indicated a characterized pattern, compared with that in the scenery stimulus.

### Validation of the SVM classifier

#### Data and analysis

A 36-fold cross-validation among the three types of categorized data (unconditioned and conditioned neutral faces and scenery) was performed to evaluate the accuracy of the SVM classifier. The tested EEG data were the same as those used in Study 1 (12 participants). An SVM classifier trained by using 35 data was evaluated by using the remaining data (i.e., test data); this procedure was repeated, with modifications to the training and test data (a round robin for all the data: 36 rounds), to calculate classification accuracy. The independent parameter γ in Equation (3) was fixed at a constant value during this cross-validation (ν = 0.5), thereby resulting in changes to the other parameters, such as the optimal objective value of the dual SVM problem and the bias term in the decision function. Here, the independent kernel parameter γ of Equation (3) was set as four types: 0.0001, 0.01, 1, and (number of features)^−1^, and the accuracy of the cross-validation was computed for each kernel parameter. The tolerance of termination criterion for the SVM was set at ε^−*6*^.

#### Classification results

**Figure 7:**
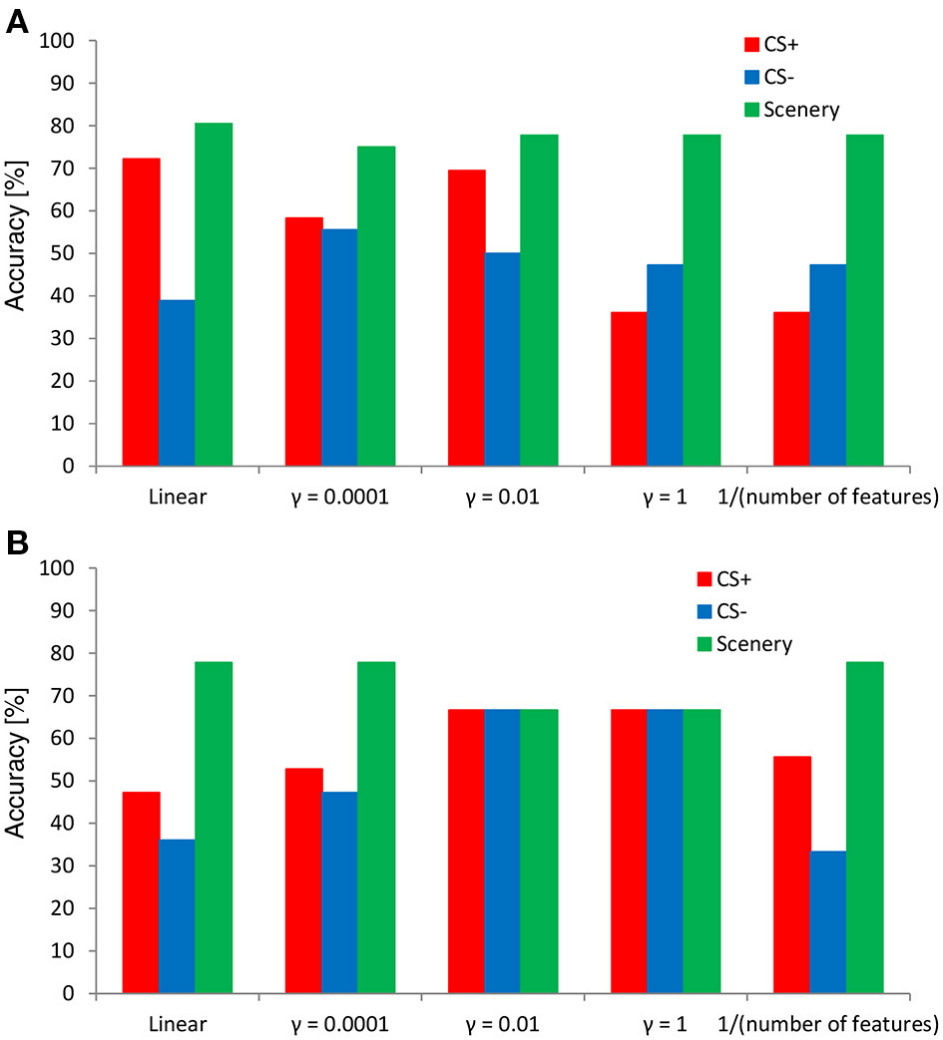
**Results of cross-validation using the SVM classifier**. The SVM features **(A)** time–course and **(B)** time–frequency data.

Figure 7 shows the results of the 36-fold cross-validation regarding the performance of the SVM classifier in evaluating the negative emotional responses to inexpressive faces determined from the ERP responses. In both features, the SVM classifier exhibited an accuracy higher (80% at the maximum) than the chance level (i.e., 33% in each). Overall, the classification accuracies for the faces and scenery tended to be higher than those for the CS+ and CS− conditions. However, classification accuracy depended on the kernel parameter value. Compared with the time–course data of the SVM classifier, the wavelet-transform data showed stable accuracy in each category (e.g., almost 70% especially in the kernel parameter γ = 0.01 and 1), indicating the existence of the optimal parameter setting.

### Morphing application

After the SVM classifier was trained by the above-mentioned training sample, the face morphing application (Microsoft Visual C++ 2012, OpenCV ver. 2.4, and LIBSVM ver. 3.17) was tested under the hypothesis of real-time data acquisition. When a neutral face associated with a negative emotion is predicted by the SVM classifier, the face morphing application is automatically triggered. As shown in Figure 5B, the target neutral face gradually (about 1–2 s) changed into a negative one (JAFFE database^1^). Therefore, individuals' emotions can be easily estimated using the proposed non-verbal communication tool.

### Discussion 2

In Study 2, the SVM classifier for non-verbal communication was evaluated by identifying the ERP responses to implicit emotional faces. For the control of mechanical or computer devices, active BMI/BCI studies have been based on the features of EEG data on various scalp regions (e.g., P300 responses) (Piccione et al., 2006; Mak et al., 2012). The present study especially focused on the activity of the posterior temporal lobe in relation to face processing; the SVM feature was used after the application of the wavelet transform. In the optimal range of the kernel parameter, the SVM classifier for the time–frequency domain showed stable accuracy (almost 70% in each category). Developing an auto-tuning method for appropriate parameter setting of the SVM classifier is an advantageous approach because of the individual differences of ERP responses. Thus, wavelet transform data will be effective for such a case. An accuracy higher than 60% will be regarded as a tentative indicator of a successful case for affective BCIs (Nijboer et al., 2009; Mak et al., 2011). A desirable future direction for BMI/BCI research is the development of small embedded systems for the everyday use of face morphing software that can reflect various emotions.

A critical safety problem arises when a patient experiences a severe accident as a malfunction occurs in active BCIs for basic motion (e.g., wheelchair, artificial arm, etc.). The proposed system is a device intended to enhance quality of life through emotional estimation. When a patient may possibly experience a negative emotion, the people around the patient (e.g., families, friends, and carers) can support him/her by paying particular attention to emotional estimation. If they recognize or predict the patient's negative emotions (e.g., 60 or 70% negative feeling, no perfect or maximum level for each emotion), the patient could live a full and humane life. Nevertheless, further studies on more accurate classifier methods are required. For example, a cascaded classifier may effectively increase classification accuracy.

Time–frequency analyses can identify the neural activities crucial for emotional face processing, but further modifications are required to obtain higher accuracy in SVM classification. This requirement may be satisfied with the direct image analysis (e.g., the bag of features scheme; Csurka et al., 2004) of contour maps in time–frequency data (Kashihara et al., 2011). Speeded-up robust features (SURF) and scale-invariant feature transform (SIFT) can effectively search for local information on object boundary (Bay et al., 2008). In the bag of features scheme, visual words are generated by the k-means algorithm to cluster the feature vectors of SIFT or SURF and create a visual vocabulary (Duda et al., 2001). Each image can be represented by a histogram of visual words. Therefore, the bag of features scheme may serve as a means of novel interpretation that determines the effective features of an SVM classifier and could extract meaningful changes in time–frequency data.

Several limitations constrain the practical application of the BCI approach adopted in this study. The specific brain region considered in the classification was limited to the right FG area identified by source localization from multiple electrodes attached onto a participant's scalp. Crucial issues for consideration are the separation of brain activities into multiple inputs and movement artifacts, which may influence the results of source localization. This study assumed application under a static measurement situation for patients and excluded active BCIs, such as moving artificial arms and wheelchairs. The next step, therefore, is to develop an accurate identifier that enables the practical application of BCIs and reduces noise; noise contamination of muscle or eye movements must be prevented through improvements in hardware, software, and algorithms.

To realize feature extraction from the frequency domain reducing external noise, data after the wavelet transform was used in machine learning (i.e., SVM); this application was implemented under the assumption that practical BCI analysis will be a component of future works. Experiment 2 was then performed to show the possibility of passive or affective BCI application for the detection of and response to high emotions. Therefore, the new techniques for wearable and hardware devices (robust to body movements) and efficient algorithms (e.g., signal filtering methods, including wavelet analysis) to eliminate external noise are desirable innovations for advancing the practical application of BCIs. In such application, accurate estimation from single trials can be factors for consideration.

The conditioned neutral facial stimuli (Experiment 1) were used to induce negative emotions based on previous experiences and to identify the specific brain area that is activated during such responses because dynamic EEG response has not been sufficiently clarified under such situations. As the next step, actual non-verbal communication should be evaluated by using real faces (families, friends, etc.) associated with patients' experiences and emotions.

Finally, the difficulty encountered in BCI training for autistic patients must be considered in the design and development of a practical system. EEG responses to facial stimuli and emotions may not require extensive training on scenarios such as those featuring active BCIs. Because posterior temporal regions are directly correlated with face processing primarily in the right FG, EEG responses might be measured in a straightforward manner, regardless of a specific training program.

## Conclusion

Inexpressive faces associated with negative experiences induce aversive and unpleasant feelings. The ERP study and source localization revealed that the aversively conditioned neutral faces activated late face processing (600–700 ms), rather than early face processing (e.g., P1 and N170), in the right FG region. Further evaluations of emotionally conditioned faces would elucidate the complicated brain activities involved in social cognition. As a non-verbal communication tool for BMI/BCIs, the proposed SVM classifier has a possibility to enable the easy detection of inexpressive faces that trigger specific individual emotions. In accordance with this classification, a face on a computer morphs into an unpleasant look. In future studies, the proposed classification method, which uses EEG signals, could be integrated with non-verbal communication tools to enable the expression of other emotions. More accurate classifiers should also be investigated to realize the practical application of BMI/BCIs.

### Conflict of interest statement

The author declares that the research was conducted in the absence of any commercial or financial relationships that could be construed as a potential conflict of interest.

## Footnotes

^1^Lyons, M. J., Kamachi, M., and Gyoba, J. (1997). *Japanese Female Facial Expressions (JAFFE), Database of digital images*. Available online at: http://www.kasrl.org/jaffe_info.html
